# Decoding Pain: A Comprehensive Review of Computational Intelligence Methods in Electroencephalography-Based Brain–Computer Interfaces

**DOI:** 10.3390/diagnostics15030300

**Published:** 2025-01-27

**Authors:** Hadeel Alshehri, Abeer Al-Nafjan, Mashael Aldayel

**Affiliations:** 1 Computer Science Department, College of Computer and Information Sciences, Imam Mohammad Ibn Saud Islamic University (IMSIU), Riyadh 11432, Saudi Arabia; 2 Information Technology Department, College of Computer and Information Sciences, King Saud University, Riyadh 11543, Saudi Arabia

**Keywords:** brain–computer interface (BCI), pain assessment, electroencephalography (EEG)

## Abstract

Objective pain evaluation is crucial for determining appropriate treatment strategies in clinical settings. Studies have demonstrated the potential of using brain–computer interface (BCI) technology for pain classification and detection. Collating knowledge and insights from prior studies, this review explores the extensive work on pain detection based on electroencephalography (EEG) signals. It presents the findings, methodologies, and advancements reported in 20 peer-reviewed articles that utilize machine learning and deep learning (DL) approaches for EEG-based pain detection. We analyze various ML and DL techniques, support vector machines, random forests, k-nearest neighbors, and convolution neural network recurrent neural networks and transformers, and their effectiveness in decoding pain neural signals. The motivation for combining AI with BCI technology lies in the potential for significant advancements in the real-time responsiveness and adaptability of these systems. We reveal that DL techniques effectively analyze EEG signals and recognize pain-related patterns. Moreover, we discuss advancements and challenges associated with EEG-based pain detection, focusing on BCI applications in clinical settings and functional requirements for effective pain classification systems. By evaluating the current research landscape, we identify gaps and opportunities for future research to provide valuable insights for researchers and practitioners.

## 1. Introduction

Pain—a complex sensory and emotional experience associated with actual or potential tissue damage—plays a crucial role in an individual’s life. This multi-dimensional experience is produced by characteristic “neurosignature” patterns of nerve impulses generated by a widely distributed neural network in the brain. Furthermore, pain substantially affects the quality of life and is subjective as it is experienced and perceived differently by different individuals. Pain assessment depends on an individual’s personal experience, which cannot be directly observed or evaluated by others. Pain experiences can be influenced by various factors, including emotions, beliefs, cultural backgrounds, and psychological states [1].

Pain assessment is a challenging task. In modern clinical pain assessment, healthcare professionals determine the pain levels of their patients through interviews [2], which are difficult for individuals who cannot communicate or verbally express their pain. Therefore, effective and objective pain detection methods are urgently required.

Brain–computer interfaces (BCIs) are communication systems that link the brain to external devices [3]. BCIs, which translate brain activity signals into the necessary output [4], have demonstrated potential in various healthcare domains such as stroke rehabilitation, mental state evaluation, and communication restoration [5]. For instance, BCIs have been instrumental in stroke recovery and rehabilitation methods, enabling targeted therapies through which patients can regain motor control and enhance their overall functional abilities [6]. By analyzing brain activity patterns, BCI systems can provide insights into cognitive processes, attention levels, and emotional states, providing information for optimizing training programs, assessing cognitive workload, or detecting mental fatigue signs [7].

In recent years, neuroscientists and neuroimaging researchers have explored the use of both EEG- and fMRI-based BCIs for objective pain detection [8]. The relevant features in raw neural signals are preprocessed, extracted, and classified using machine learning (ML) algorithms. ML techniques are used to perform tasks such as filtering for signal processing and spectral analysis for feature extraction. Supervised ML models identify and classify pain by learning from labeled data [9,10,11,12], and deep learning (DL) models can automatically extract meaningful features from raw neural signals without manual feature engineering. In addition, DL models can capture spatial patterns through stacked hidden layers and classify pain based on neural data [13,14].

ML has emerged as a key branch of artificial intelligence (AI), as it offers a diverse range of symbolic and statistical approaches for analyzing and interpreting neural data. ML techniques empower systems to learn and refine themselves through experience by utilizing computational models to generate predictions or decisions without requiring explicit programming. In the context of BCIs, ML plays a critical role in various applications, revolutionizing the field by improving the diagnosis of various diseases and sleep disorders, predicting epileptic seizures, and facilitating rehabilitation [15]. DL is a specialized field within ML that utilizes extensive data and multiple neural network layers to enhance its learning capabilities. In essence, DL can be viewed as a powerful model within the ML domain that is inspired by the intricate workings of the human brain. Furthermore, both DL and ML are subsets of AI [16]. DL can uncover complex patterns and relationships within data, resulting in improved accuracy and decision-making for AI models. DL appears to be effective in areas such as natural language processing (text), automatic speech recognition (audio), computer vision (image), and intrusion detection [16]. In the context of BCIs, AI plays a critical role in pain decoding, enhancing the ability to interpret neural signals associated with pain perception. Techniques such as support vector machines, random forests, and neural networks have revolutionized the field, leading to improved diagnostic capabilities and personalized pain management strategies. For instance, recent studies have demonstrated that ML algorithms can accurately classify pain-related brain activity, significantly contributing to our understanding of pain mechanisms and offering new avenues for therapeutic interventions [17].

The present research reviews the literature on pain detection based on electroencephalography (EEG) signals. We examine 20 published peer-reviewed articles focusing on ML and DL approaches for EEG-based pain detection. We also comprehensively overview the advancements and challenges related to EEG-based pain detection using ML and DL methodologies. The aim is to provide researchers and practitioners with insights into the current and future trends of this field. We then explore the use of BCIs in clinical settings and discuss the functional requirements and computational processing constraints of pain classification systems. By examining the current state of research, we aim to identify gaps, challenges, and opportunities for future research in the field.

The remainder of this paper is structured as follows: The background is presented in Section 2. Section 3 explains the method used in this review. Section 4 reviews the studies on pain assessment systems. Section 5 covers the results and discussion. Section 6 provides insights for future research. This review concludes with Section 7.

## 2. Background

This section presents the foundational background of our research, focusing on BCIs as our key research concept. Understanding the concepts involved in BCI technology is necessary for comprehending the objectives and importance of our research.

BCIs are a rapidly advancing technology facilitating direct communication between human brains and external devices. The use of BCI signals from the brain can potentially transform various domains, including healthcare, rehabilitation, and human–computer interactions. This section explores the fundamental principles of BCIs, including their operational mechanisms, methodologies for signal acquisition, and diverse applications in healthcare contexts.

### 2.1. Brain–Computer Interface

**Figure 1:**
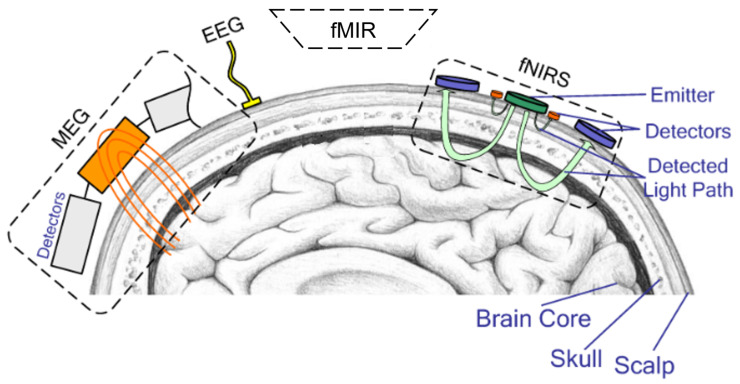
Placement of different BCI sensors: magnetoencephalography (MEG), electroencephalography (EEG), near-infrared spectroscopy (fNIRS), and functional magnetic resonance imaging (fMRI).

BCI is a communication system through which the brain can communicate with an external device. BCIs can be classified into three categories: non-invasive BCIs (in which the sensors are placed on the scalp over the skin), semi-invasive or partially invasive BCIs (in which the sensors are placed on the brain surface beneath the skin to evaluate electrical activity), and invasive BCIs (in which microelectrodes are directly implanted into the brain during surgery to evaluate the activities of single neurons) [7]. Non-invasive BCI-controlled interface methods include EEG, magnetoencephalography (MEG), functional near-infrared spectroscopy (fNIRS), and functional magnetic resonance imaging (fMRI), as illustrated in Figure 1.

EEG—the most commonly used and oldest non-invasive brain-imaging technique—detects brain signals related to neurological diseases, seizure disorders, sleep disorders, other symptoms, pain, and various stress-induced problems [18]. As EEG can be used to investigate and track pain biomarkers [19], it is selected to obtain input brain signals to BCIs in our research. EEG is employed to evaluate electric potentials produced by different regions of the brain. It involves evaluating the electrical activities of neurons with metal electrodes placed on the scalp [5].

Neural activities are either rhythmic or transient [20]. Rhythms, or brainwaves, are repetitive forms of neural activity with different frequency bands denoted as delta, theta, alpha, beta, gamma, and mu rhythms. Transient activities replicate the action potentials of certain neurons with spikes, which can be recognized by their amplitude, frequency, position, shape, recurrence, and operational properties. The common types of transient activities are event-related potentials (ERPs) and event-related spectral perturbations [20].

**Figure 2:**
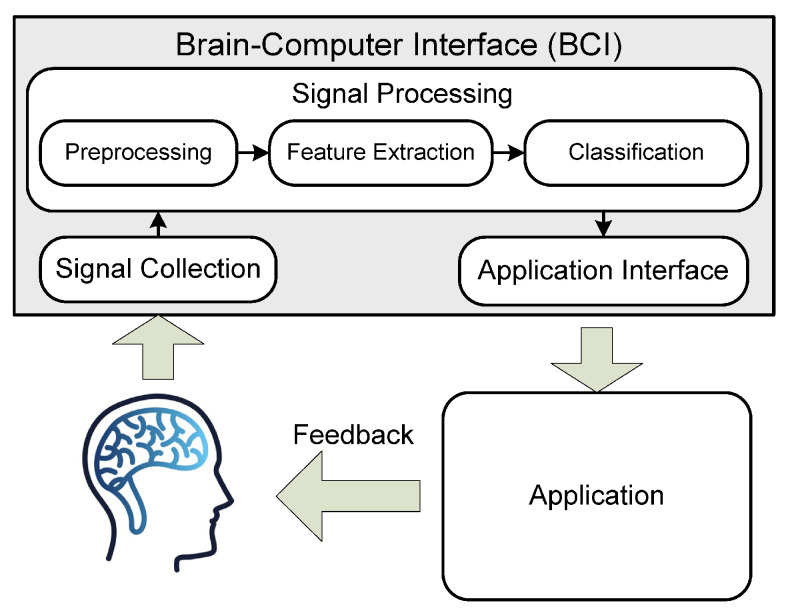
Primary stages of a BCI system.

A BCI system proceeds through five stages (Figure 2) [21]: data collection or acquisition, signal preprocessing, feature extraction, classification, and application interface. These stages are outlined below.

1. Data collection: The signal data (cerebral neuron activities) are recorded by electrodes positioned on the scalp of the subject. The electrode placements are governed by certain protocols based on the neuroscientific findings [18].
2. Preprocessing: The acquired signals are monitored and enhanced through signal filtering, signal cutting, amplitude scaling, verification of expert marks, artifact detection, noise detection and removal, and signal segmentation [18].
3. Feature extraction: A feature is a unique data characteristic. Feature extraction derives new features from the existing ones to reduce the measurement costs and enhance the classifier performance. Recent BCI-based studies [18] have extracted the features from EEG signals using linear or nonlinear feature extraction methods. Feature extraction can be performed in the time, frequency, time–frequency, or spatiotemporal frequency domains. The selections of feature extraction method and feature domain are typically guided by neuroscientific principles in the related domain [18].
4. Classification: Also referred to as the translation algorithm, the classification algorithm translates the extracted signal features into device commands. Translation algorithms are developed in ML classifiers [22].
5. Application interface: The application interface is the final stage of feedback processing. In pain detection, the expected feedback is the intensity or presence of pain.

### 2.2. AI Model Evaluation

This section presents the evaluation measurements employed by recent studies to assess the performance and effectiveness of the BCI pain detection system. To evaluate the performance of machine learning models in the context of pain detection and rehabilitation, it is essential to utilize a variety of performance metrics. Conventional metrics such as the F1 score and recall are pivotal in assessing model efficacy, particularly in situations where class imbalance may skew results. The recall metric in Equation (1) measures the percentage of correctly classified instances of the pain level [23]. The F1 score in Equation (2), defined as the harmonic mean of precision and recall, provides a balanced measure of a model’s accuracy in identifying positive cases [23]. The precision metric in Equation (3) focuses on the quality of accuracy and measures how closely the model’s classifications align with each other, regardless of their accuracy [23]. The accuracy metric in Equation (4) refers to the degree to which the model’s measurement results correspond to the correct values. This indicates how close the measured values are to known or standard values [23].

(1) $$\mathrm{Recall}=\frac{TP}{TP+FN}$$

(2) $$F-\mathrm{measure}=\frac{2\times \mathrm{Precision}\times \mathrm{Recall}}{\mathrm{Precision}+\mathrm{Recall}}$$

(3) $$\mathrm{Precision}=\frac{TP}{TP+FP}$$

(4) $$\mathrm{Accuracy}=\frac{TP+TN}{TP+TN+FP+FN}$$

In addition to these conventional metrics, it is important to consider quantitative evaluation indicators specific to the rehabilitation field. Metrics such as accuracy, area under the receiver operating characteristic curve (AUC-ROC), and specificity are crucial for understanding a model’s capability in distinguishing between painful and non-painful conditions. Furthermore, mean absolute error (MAE) and mean squared error (MSE) are valuable in assessing the accuracy of pain level predictions in regression contexts.

### 2.3. EEG-Based BCI in Healthcare Applications

In the healthcare industry, EEG-based BCI systems can be employed to detect complex disorders such as epileptic seizures, Alzheimer’s disease, and sleep disorders in EEG data [24].

Sleeping disorders can be identified by monitoring the spontaneous EEG signals during sleep. Sors et al. [25] adopted a convolutional neural network (CNN) for the sleep stage classification of single-channel EEG data, achieving an overall multiclass classification accuracy of 87%. Biswal et al. [26], who detected sleep disorders using a recurrent neural network (RNN), reported an average accuracy of 85% in sleep stage annotation.

DL models can classify and diagnose neurological disorders, especially epileptic seizures, with high efficacy. In a study of epileptic spike detection [27], a CNN achieved a higher area under the curve (AUC) of the receiver operating characteristic (0.947) than other classifiers such as state vector machines (SVMs). A 13-layer CNN model achieved an accuracy of 88% in depression diagnosis [28] and a CNN–RNN hybrid method achieved a specificity of 90.37% in seizure detection [29].

Roy et al. [30] proposed ChronoNet—a hybrid model comprising one-dimensional (1D) convolution layers and an RNN architecture—for classifying normal and abnormal brain activities. Designed for efficient processing of EEG data in clinical settings, ChronoNet is formed by stacking multiple 1D convolution layers followed by deep gated recurrent unit (GRU) layers. Each 1D convolution layer employs multiple filters of exponentially varying lengths, and the stacked GRU layers are densely connected in a feedforward manner. This architecture easily captures the patterns emerging at different scales in the time domain and mitigates vanishing gradients. ChronoNet detected normal and abnormal activities with an accuracy of 85% [30].

## 3. Research Methodology

Objective pain assessment is a relatively new field wherein the involved assessment has been performed using various technologies. Among different modalities, BCI technology is a promising approach for measuring pain indicators, as demonstrated in numerous journal and conference publications [9,10,11,12].

For the present review, we conducted a comprehensive search of the Web of Science (WoS) database to assess the impact and quality of EEG-based brain–computer interface (BCI) research on pain detection from 2017 to 2024. The WoS provides access to a variety of citation databases, including the IEEE/IEE Library, Springer Link Online Libraries, Science Direct (Elsevier), and the ACM Digital Library.

Our search focused on identifying peer-reviewed papers indexed by the Institute for Scientific Information (ISI). Utilizing the WoS database allowed us to ensure comprehensive coverage of the relevant literature in the field of BCI-based pain detection. The inclusion of peer-reviewed ISI papers enhances the rigor and reliability of our review, as these studies have undergone a thorough peer-review process.

We specifically selected the timeframe of 2017 to 2024 to emphasize the most recent trends and methodologies in EEG-based pain detection. While we acknowledge the significance of research published prior to 2017, our primary objective was to capture the latest advancements in this rapidly evolving field. Given the substantial increase in publications on this topic in recent years, we believe that focusing our review on more recent studies facilitates a clearer and more relevant analysis, avoiding the potential distraction of an extensive volume of earlier works.

The search keywords included “pain”, “EEG”, and “BCI”, and the search strategy was formulated as (Pain AND (EEG OR Electroencephalography) AND (BCI OR Brain- Computer Interface)).

**Figure 3:**
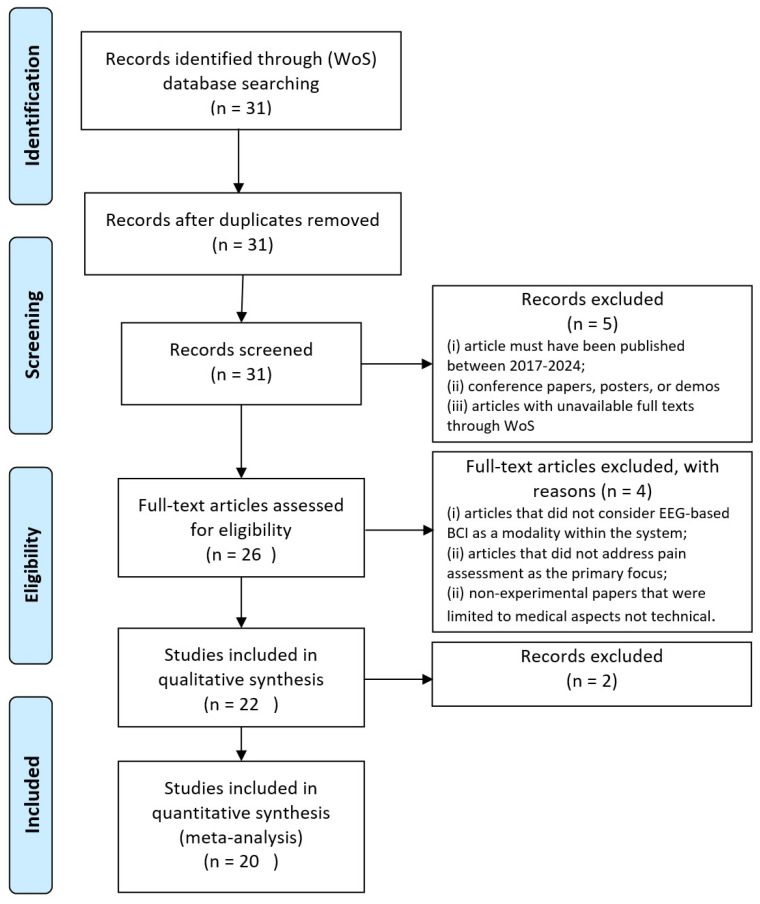
Article filtering process.

The initial search yielded 31 articles satisfying the selection criteria of our first filtering process. Our research methodology, review process, and filtering procedures (Figure 3) followed the Preferred Reporting Items for Systematic Reviews and Meta-Analyses (PRISMA) guidelines [31]. To pass the first filtering process, the article must (i) have been published between 2017 and 2024; (ii) not be a conference paper, poster, or demonstration, which typically lack sufficient content for a thorough evaluation; and (iii) have full texts available through WoS.

Twenty-six articles remained after the initial revision cycle. During the second screening procedure, we manually reviewed each paper under further exclusion criteria. Publications meeting one or more of the following conditions were excluded: (1) articles not including EEG-based BCI as a modality within the system, (2) articles in which pain assessment was not the primary focus or a substantial contributor, and (3) nonexperimental papers limited to medical aspects without technical contributions.

After scanning the titles, abstracts, keywords, and conclusions during the second revision procedure, 22 articles remained. Finally, we read the full texts of these articles for a more detailed screening. During the third revision cycle, we eliminated two additional articles under the previously established exclusion criteria, resulting in 20 eligible articles for the present review.

**Figure 4:**
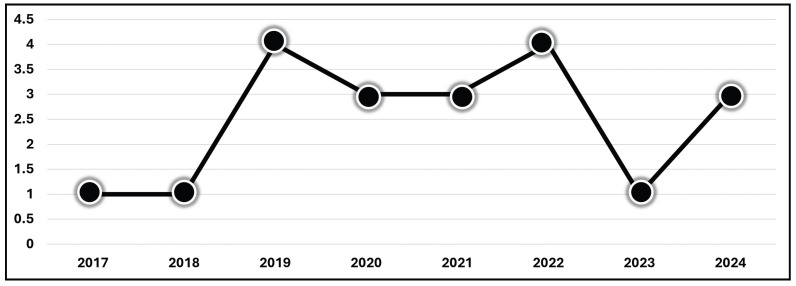
Timeline of the reviewed studies. The vertical axis plots the number of publications in each year.

The 20 eligible peer-reviewed articles were divided into two categories: ML and DL approaches. All articles were published between 2017 and 2024 (Figure 4). We extracted the relevant data (EEG datasets, EEG types, pain classes, computational methods, and accuracy metrics) from each study.

## 4. Literature Review

This section first reviews the studies on pain assessment systems employing traditional ML algorithms such as support vector machines (SVMs), k-nearest neighbors (KNN), and random forests (RFs). The aim is to understand the methodologies and features of pain assessment and to determine the performance and limitations of traditional machine learning approaches.

In contrast, we then review studies applying DL techniques in pain assessment systems, which leverage complex architectures and high-dimensional data to automatically extract features, often resulting in improved classification performance. While ML methods typically require manual feature extraction and are limited in their ability to model complex relationships, DL approaches excel in capturing intricate patterns within the data, thereby enhancing predictive accuracy.

Through this analysis, we can comprehend the advancements, challenges, and future directions of both ML and DL in pain assessment, highlighting how these methodologies complement each other in the context of brain–computer interfaces (BCIs) for pain detection.

The ML and DL studies reviewed herein are summarized in Table 1 and Table 2, respectively. Each table describes the EEG dataset, EEG signal type, number of classes considered, and accuracy metrics employed.

### 4.1. EEG-Based Pain Detection Using Traditional ML Approaches

This section reviews the use of traditional ML approaches for pain detection in EEG signals. As shown in Table 1, different ML techniques for EEG-based pain assessment adopt different feature extraction and classification methods. Modares-Haghighi et al. [9] quantified different pain classes through binary classification and three-class classification. They assessed the pain levels of 23 subjects from the evaluated EEG signals and self-reported pain levels of the participants during a cold pressor test. The data were collected twice for each participant. The method of Modares-Haghighi et al. [9] generates a brain graph matrix for each pain level, identifies the location of pain in each class, selects the discriminant features through sequential forward feature selection, and finally classifies the data using a multilayer SVM. The accuracy of their method was 92% on binary classification (pain vs. no pain) and 89% on three-class classification (low, moderate, or high pain intensity).

Nezam et al. [10] proposed a five-class pain detection method that distinguishes between no pain and four levels of pain intensity: low, medium, high, and very high. They collected the EEG recordings from 24 healthy subjects during a cold pressor test. Their method involves first computing the grand-average brain maps over the alpha and delta bands at each pain level and then selecting the discriminant features (Shannon, approximate, and spectral entropies) through sequential forward feature selection. Finally, the results of the KNN and SVM classifiers are compared, achieving an accuracy of 83%.

The automated assessment method of Bonotis et al. [11] similarly identifies five pain levels: no, low, medium, high, and unbearable pain. The authors recorded the EEG signals of 22 healthy volunteers undergoing a cold pressor test. Their method extracts the band power features from EEG windows and then classifies the signals using a stochastic forest (SF) ensemble learning algorithm, achieving an accuracy of 72%.

Alazrai et al. [12] investigated a similar system for pain detection. They recorded the EEG signals of 24 participants who reported their own pain levels under a tonic cold pain stimulus. The features of the EEG signals were extracted from a quadratic time–frequency distribution and classified with an SVM classifier, achieving an 83.4% accuracy.

Vijayakumar et al. [32] classified 10 pain intensity levels using a characterization model. They collected the EEG data of 25 healthy subjects under a tonic thermal pain stimulus and assigned them to pain scores ranging from 1 to 10. Their model extracts the time–frequency representations of the EEG signals model using Gabor wavelets and classifies the features using an RF classifier. They reported an accuracy of 89.45%.

Sun et al. [33] applied unsupervised ML in an algorithm that detects the onset of pain signals and statistically compares the ERPs of EEG signals. They recorded a neural dataset of ERPs collected from rats subjected to mechanical noxious stimulations. They also collected the local field potentials from the cortical regions involved in pain processing. An algorithmic pain decoder and ML automate the detection of pain onset through the involved BCI. The authors concluded that pain-triggered neural activity first changes in the primary somatosensory cortex and then in the anterior cingulate cortex.

Alazrai et al. [34] implemented a tonic cold pain detection algorithm that simulates pain experiments with a tonic pain simulator. They recorded a dataset of EEG signals from 24 healthy subjects under tonic cold pain stimuli, extracted the quadratic time–frequency distributions from the EEG signals, and analyzed them using the Choi–Williams distribution. Finally, they classified the data into pain and no pain classes using an SVM, achieving an accuracy of 89.24%.

Afrasiabi et al. [35] proposed a hierarchical classification strategy that differentiates among five pain intensity levels: no, low, medium, high, and intolerable pain. They recorded the EEG signals of 44 subjects undergoing a cold pressor test and annotated them with the five pain levels. They extracted the informative features from the EEG signals through sequential forward selection and then trained a Bayes-optimized SVM classifier at each decision node of the hierarchy. The accuracy of their algorithm was 99.8% on binary classification (pain vs. no pain) and 93.33% on five-class classification.

Zolezzi et al. [36] presented a pain severity detection system based on linear and nonlinear EEG features. They collected the EEG data of 35 neuropathic patients who had reported their individual pain experiences during daily activities on a questionnaire. To extract the linear and nonlinear features, they computed the absolute band power in each frequency band and the approximate entropy of each channel, respectively. Employing a multilayer SVM classifier, they finally classified the features into three pain severity levels (low, medium, and high) with an accuracy of 96%.

Sai et al. [37] objectively identified pain during the first stage of labor using continuous EEG signals and an SVM classifier. They collected the EEG and cardiotocography data of 10 women undergoing the first labor stage. Their classifier distinguished between pain and no pain with an accuracy of 84%.

Zhang et al. [38] identified pain-discriminant features using laser-evoked brain potentials (LEPs) and least absolute shrinkage and selection operator (LASSO) regression. LEPs are ERPs elicited after stimulating the skin with lasers. They recorded the EEG and fMRI data of 366 participants receiving stimuli across four sensory modalities and calculated the AUC, demonstrating that LEP features are pain-selective and cannot track tactile, auditory, or visual discriminates.

Using a publicly available EEG dataset collected from 36 participants, Tasci et al. [39] investigated chronic neuropathic pain in patients already experiencing varying levels of pain as part of their condition. Using the Brief Pain Inventory (BPI), the researchers grouped the patients into three pain levels (low, moderate, and high) based on the patients’ self-reported pain severity. They then developed a feature extraction function called the black–white hole pattern (BWHPat). Inspired by astronomical phenomena, the BWHPat function dynamically selects the most suitable pattern among 14 options. The textural and statistical features and a tunable q-factor wavelet transform (TQWT) were also incorporated for multi-leveled feature extraction. Feature selection was performed via iterative neighborhood component analysis, and channel-specific results were obtained using a KNN classifier. The accuracy of the BWHPat-driven model reached 99% across the three classes.

**Table 1:** Recentstudies using the ML technique for pain detection with EEG.

Reference	EEG Dataset	EEG Type	Classes of Pain	ML Methods	Accuracy
Modares-Haghighi et al., 2021 [9]	Collected from 23 subjects via cold simulation	Rhythms (alpha band)	5 (no, low, mid, high, and intolerable pain)	SVM	89%
Nezam et al., 2018 [10]	Collected by 24 subjects through cold simulation	Rhythms (alpha band)	5 (no pain and first, second, third, and fourth levels of pain)	KNN and SVM	83%
Bonotis et al., 2019 [11]	Collected from 22 subjects via cold simulation	Rhythms (five-frequency band: gamma, delta, theta, alpha, and beta)	5 (no, low, mid, high, and unbearable pain)	SF	72.7%
Alazrai et al., 2019 [12]	Collected from 24 subjects through cold simulation	Rhythms (gamma, delta, theta, alpha, and beta)	2 (no pain and pain)	SVM	83%
Vijayakumar et al., 2017 [32]	Collected from 25 subjects via thermal simulation	Rhythms (five-frequency band: gamma, delta, theta, alpha, and beta)	10 (pain range)	RF	89%
Alazrai et al., 2019 [34]	Collected from 24 subjects through cold simulation	Rhythms (five-frequency band: gamma, delta, theta, alpha, and beta)	2 (no pain and pain)	SVM	89%
Afrasiabi et al., 2021 [35]	Collected from 44 subjects via cold simulation	Rhythms (alpha band)	5 (no, low, mid, high, and intolerable pain)	Bayes-optimized SVM	93%
Zolezzi et al., 2021 [36]	Collected from 35 patients based on questionnaire monitoring	Rhythms (five-frequency band: gamma, delta, theta, alpha, and beta)	3 (low, mid, and high pain)	Multilayer SVM	96%
Sai et al., 2019 [37]	Collected from 10 parturient women during labor	Rhythms (delta, theta, alpha, and beta bands)	2 (no pain and pain)	SVM	84%
Zhang et al., 2022 [38]	Collected from 366 subjects using four simulations	ERP (peak amplitude and latency’s LEP)	2 (low and high pain)	LASSO	AUC Curve
Tasci et al., 2024 [39]	Collected from 36 chronic patients recorded in a public dataset using BPI tools	Rhythms (alpha, beta, gamma, or theta)	3 (low, moderate, and high pain)	KNN	99%
Leng et al., 2024 [40]	Collected from 26 subjects participated through laser-based simulation	Rhythms (alpha, beta)	5 (pain0, pain1, pain2, pain3, and pain4)	SVM and KNN and RF	89%

Leng et al. [40] assessed pain levels from the EEG data of 26 participants during a laser-stimulated pain experiment. They cleaned the EEG signals using a suite of preprocessing techniques—independent component analysis (ICA), a trapezoidal filter, and a bandpass filter—and then applied the Stockwell transform on the preprocessed EEG data to extract the time–frequency features. They also augmented the data with synthetic EEG data generated by a generative adversarial network (GAN) with gradient penalty. For the pain level classification task, they evaluated the SVM, RF, and KNN classifiers. The different models achieved an average classification accuracy of 89% on five pain levels (pain0, pain1, pain2, pain3, and pain4).

### 4.2. EEG-Based Pain Detection Using DL Approaches

DL can learn the hierarchical representations of EEG signals in pain detection. Table 2 summarizes the different DL approaches for processing EEG signals, along with their feature extraction and classification methods. The review of Gkikas and Tsiknakis [41] highlights the growing use of DL in pain assessment. Two studies [42,43] focused on pain assessment from EEG signals in their reviews.

Yu et al. [42] introduced an EEG-based CNN model that accurately classifies tonic cold pain states. They recorded the EEG data of 32 subjects under cold stimulus conditions and classified them into no, moderate, and severe pain. To learn the temporal representations in the EEG data, they extracted several bands from the biological signals (alpha, beta, and gamma), providing diverse frequency band-based inputs, and applied a convolution module to each band. The accuracy of their model was 97%.

Wang et al. [43] assessed pain from the EEG potentials of 29 subjects. They proposed an autoencoder model that encodes the raw EEG data into a compressed format, enabling effective feature extraction. The extracted features were then fed into a logistic regressor classifier. High and low pain were categorized with an accuracy of 74%.

Chen et al. [13] proposed a neural network algorithm that classifies raw EEG data into two classes (pain and no pain). They collected the EEG signals from 10 chronic-back-pain patients during movement and video stimulation. Their algorithm filters the EEG data, extracts the EEG features, and then classifies them using the neural network, achieving an 83% accuracy.

Elsayed et al. [14] objectively quantified pain perception through a combination of data generation techniques and DL models. They collected the EEG signals of 30 participants during a cold pressor test. Their approach first extracts the pain index matrix (PID) from the EEG signals in the alpha band. The PID, which measures the average amount of power in the alpha band, is then incorporated with noise and fed to a variational autoencoder (VAE) to generate new data. The generated data are fed to an artificial neural network that classifies pain into four levels (no pain, low pain, moderate pain, and high pain) with an accuracy of 94.83%.

Wu et al. [44] introduced the adversarial reconstruction CNN, a DL-based method that learns the invariant EEG representations for accurate pain intensity assessment. They collected the EEG data of 24 participants undergoing the cold pressor test and classified them into four levels of intensity: no, low, high, and intolerable pain. They converted the EEG signals into multispectral topography maps (delta, beta, and alpha) and simultaneously processed the EEG signals using a CNN and RNN to fuse the spatial and temporal features. The four pain levels were classified with an accuracy of 92%.

Applying CNNs, Han et al. [45] classified pain experiences based on the phase connectivity in the alpha frequency band of EEG signals. The EEG signals were recorded from 36 participants under thermal and resting state conditions. The extracted features were re-organized into square matrices to fit the input requirements of CNNs. The proposed CNN classifier distinguished between pain conditions and eye-open resting states with an accuracy of 94.16%.

Fu et al. [46] proposed a spatiotemporal DL framework for scalp (EEG)-based automated pain assessment in children. The dataset comprises the scalp EEG data of 33 pediatric patients under a pain stimulus (arterial puncture). The EEG signals were preprocessed through a bandpass filter, a notch filter, and ICA. The authors also applied two-electrode reduction plans to align with clinical findings, along with several feature extraction methods to obtain the frequency domain features, time domain features, and nonlinear entropy. The authors combined three-dimensional hand-crafted features and fed them into a DL model called the spatiotemporal pain assessment network, which integrates a transformer and a CNN. The accuracy of pain recognition (pain or no pain) was 87.83%.

**Table 2:** Recent DL-based studies of pain detection from EEG data.

Reference	EEG Dataset	EEG Type	Classes of Pain	DL Method	Accuracy
Yu et al., 2020 [42]	Collected from 32 subjects via cold simulation	Rhythms (gamma, alpha, and beta bands)	3 (no , moderate, and severe pain)	CNN	97%
Wang et al., 2020 [43]	Collected from 32 subjects	-	2 (high and low pain)	Autoencoder and logistic regressor	74%
Chen et al., 2022 [13]	Collected from 10 patients through movement and video stimulation	Rhythms (gamma, delta, theta, alpha, and beta)	2 (no pain and pain)	CNN	83%
Elsayed et al., 2020 [14]	Collected from 30 subjects via cold simulation	Rhythms (alpha band)	4 (no, low, mid, and high pain)	VAE and NN	94%
Wu et al., 2022 [44]	Collected from 24 subjects through cold simulation	Rhythms (alpha, beta, and delta bands)	4 (no, low, high, and intolerable pain )	CNN and RNN	92%
Han et al., 2022 [45]	Collected from 36 subjects via thermal and resting state simulation	Rhythms (alpha band)	2 (pain and no pain)	CNN	94%
Fu et al., 2024 [46]	Collected from 33 pediatric patients using arterial puncture as a pain stimulus	Rhythms (delta band)	2 (pain and no pain)	CNN	87.83%

## 5. Results

This section analyzes and discusses recent pain studies in terms of EEG type, pain type, number of participants, simulator type, and computational methods of the ML and DL approaches.

### 5.1. EEG Type

Table 3 and Table 4 summarizes the various EEG types examined in pain detection studies. The most commonly observed EEG features were the energies of EEG signals in the alpha band, which were analyzed in five studies [9,10,14,35,45]. One study specifically investigated the delta band [46].

Other studies investigated combinations of bands. One study explored the alpha and beta bands [40] and another focused on the gamma, alpha, and beta bands [42]. The alpha, beta, and delta bands were studied in [44]. Analyses of four bands, specifically, the delta, theta, alpha, and beta bands in [37] and the alpha, beta, gamma, and theta bands in [39], were also found. Finally, two studies investigated all five bands, namely, the gamma, delta, theta, alpha, and beta bands [11,36]. One study focused on ERPs, specifically analyzing the peak amplitudes and latencies of LEPs [38]. This comprehensive overview highlights the diverse EEG features utilized in pain research and underscores their importance for understanding pain mechanisms.

**Table 3:** Pain detection studies sorted by EEG type.

EEG Type	Number of Bands	EEG Band	Number of Studies	References
Rhythms	1 band	Alpha band	5	[9,10,14,35,45]
		Delta band	1	[46]
	2 bands	Alpha and beta bands	1	[40]
	3 bands	Gamma, alpha, and beta bands	1	[42]
		Alpha, beta, and delta bands	1	[44]
	4 bands	Delta, theta, alpha, and beta bands	1	[37]
		Alpha, beta, gamma, and theta bands	1	[39]
	5 bands	Gamma, delta, theta, alpha, and beta bands	5	[11,13,32,34,36]
Event-related potentials	-	(Peak amplitude and latency’s LEP)	1	[38]

**Table 4:** Pain detection studies sorted by pain type.

Pain Type	Number of Studies	References
Pain in healthy subjects	17	[9,10,11,12,14,32,33,34,35,37,38,40,42,43,44,45,46]
Chronic patient pain	1	[13]
Neuropathic pain	2	[36,39]
Labor pain	1	[37]

### 5.2. Pain Type

The reviewed studies encompassed various pain types, reflecting the diverse nature of pain assessment in EEG-based research. Most of the studies recorded the pain levels of healthy participants [9,10,11,12,14,32,33,34,35,37,38,40,42,43,44,45,46]. Other studies investigated chronic pain, particularly in patients with long-term conditions [13], neuropathic pain, particularly nerve-related pain [36,39], and childbirth, which produces a unique pain experience [37]. This diversity of pain types emphasizes the need for tailored approaches in pain detection and classification.

### 5.3. Number of Participants

Increasing the size and diversity of the participant pool enhances the statistical power and reliability of a study. Table 5 summarizes the distributions of participant counts across various experimental studies on humans. Eight studies included between 20 and 29 participants, and seven studies recruited between 30 and 39 participants. The median number of participants across all studies was 24. Understanding the effect of participant size is crucial for evaluating the statistical power and reliability of the findings.

**Table 5:** Pain detection studies sorted by number of participants.

Participant Range	Number of Studies	References
10–19	2	[13,37]
20–29	8	[9,10,11,12,32,34,40,44]
30–39	7	[14,36,39,42,43,45,46]
>40	2	[35,38]

### 5.4. Stimulus Type

Table 6 shows the percentage distributions of the different pain-inducing simulators utilized in the studies. Most studies (60%) applied the cold simulator, which uses ice as a pain stimulus. Other studies (20%) applied thermal simulators such as laser devices, questionnaires or pain assessment tools such as the BPI (13%), or a labor simulator, which likely invokes a surgical or labor-like pain experience (7%). Questionnaire-type studies obtain the subjective pain experiences of the participants rather than the EEG data under an external pain simulator. The diversity of simulator methods reflects the researchers’ goals of capturing different pain responses. The questionnaires were mostly used for chronic pain identification, whereas studies using pain simulators [36,39] analyzed the EEG data of chronic neuropathic patients who were already experiencing varying pain levels as part of their condition.

**Table 6:** Pain detection studies sorted by type of pain simulators.

Simulation	Number of Studies	Percentage	References
Cold simulators	9	60%	[9,10,11,12,14,34,35,42,44]
Thermal simulators	3	20%	[32,40,45]
Questionnaires (BPI)	2	13%	[36,39]
During labor	1	7 %	[37]

### 5.5. ML and Computational Methods

Table 7, Table 8, Table 9 and Table 10 summarize the different computational techniques used in recent ML- and DL-based studies of pain classification. These methods, including EEG signal preprocessing, feature extraction, feature selection, data generation, and classification, are discussed in the following paragraphs.

#### 5.5.1. Signal Processing

Table 7 presents the signal processing approaches utilized in EEG-based pain detection, highlighting their prevalence across multiple studies. The bandpass filter emerged as the most frequently employed method (nine studies), indicating the wide acceptance of isolating the pain-associated frequency bands. The ICA technique for artifact and noise removal was also employed in nine studies, underscoring the ability of this technique to enhance the signal quality of pain. Other studies adopted a notch filter (one study), wavelet-based artifact removal (WMA) (one study), a bandwidth filter (one study), and artifact removal via adaptive signal reconstruction (ASR) (two studies). Apparently, WMA, ASR, and notch and bandwidth filters are limitedly applied in the context of pain detection, whereas bandpass filtering and ICA are consensually regarded as effective techniques for optimizing the EEG signals in pain assessment.

As shown in the table, different signal processing methods were used in the reviewed studies. While these methods significantly enhance the quality of EEG data in BCI-based pain detection systems, their effectiveness depends on the careful implementation and consideration of the specific characteristics of the signals being analyzed. Each method has distinct advantages and disadvantages.

**Table 7:** Signal processing approaches used in EEG-based pain detection.

Method	Number of Studies	References
Bandpass filter	9	[10,12,13,14,32,34,35,36,40]
Bandwidth filter	1	[12]
Notch filter	1	[46]
Sampling	2	[13,32,34]
Artifact removal by ICA	9	[9,13,32,34,35,36,37,40,46]
Artifact removal by ASR	2	[35,36]
Artifact removal by WMA	1	[37]

For instance, bandpass filters effectively isolate relevant brain activity while eliminating irrelevant noise, thereby enhancing the signal-to-noise ratio but potentially inadvertently distorting the edges of the filtered signals if not carefully designed [10,12,13,14,32,34,35]. Bandwidth filters can enhance specific signal features; however, they may also risk excluding important information if the bandwidth is not well optimized [12].

Notch filters are removing specific frequency interferences on determined hertz power line noise, effectively cleaning the signal and improving analysis accuracy. However, excessive use of notch filters can lead to distortion of the EEG signal and potential loss of useful information at the filtered frequency [46]. Sampling is critical for converting continuous EEG signals into discrete data, allowing for efficient processing and analysis. While appropriate sampling rates can capture relevant brain activity accurately, suboptimal rates may lead to aliasing or loss of important temporal information [13,32,34]. In terms of artifact removal, ICA is separates mixed signals into their constituent components, effectively identifying and removing artifacts such as eye blinks or muscle movements. While ICA is effective, it relies on the assumption that the underlying sources are statistically independent, which may not always hold true [9,34,35,36,37,40,46].

On the other hand, ASR is a method that reconstructs clean signals by estimating the subspace of artifacts, providing robust performance against non-stationary noise. However, ASR requires careful parameter tuning and may introduce artifacts if the model is not well calibrated [35,36]. WMA is another sophisticated method for artifact removal, offering a time–frequency approach that effectively captures transient features and can separate noise from the underlying EEG signal. While WMA is handling non-stationary signals, it can be computationally intensive and may require careful selection of wavelet parameters to avoid misinterpretation of the data [37].

#### 5.5.2. Feature Extraction

Feature extraction is a core process in a BCI system. The feature extraction techniques identified in this review were wavelet transformation [32,39], a Babor transform for extracting the time–frequency features [32], a TQWT for multi-level feature extraction [39], a time–frequency analysis and quadratic time–frequency distribution for feature extraction [12,13,14,37,40], and a principal component analysis for extracting the important features [37].

Some studies [14,34,35] statistically analyzed the frequency power band of EEG signals. Other studies [9,10,35,36,40] extracted the frequency features using different entropy measures such as the Shannon entropy, approximate entropy, and spectral entropy. Two studies [12,34] constructed a time–frequency representation of the EEG signals using the Choi–Williams distribution to capture the energy of the signals. Recent studies have employed different feature selection techniques, including sequential forward feature selection [9,35].

**Table 8:** Feature extraction approaches used in EEG-based pain detection.

Method	Number of Studies	References
Time–frequency domain	5	[12,13,14,37,40]
Frequency feature (different entropy and PSD)	5	[9,10,35,36,40]
Wavelet transformation	2	[32,39]
LEP feature (different peak)	1	[38]
Statistical analysis of power band	4	[14,34,35]
Principle component analysis	1	[37]
Choi–Williams distribution	2	[12,34]

As shown in the table, various feature extraction methods were used in the reviewed studies, each offering distinct advantages and disadvantages that affect the performance of pain detection systems. These strengths and weaknesses should be considered when selecting feature extraction methods in BCIs to optimize the analysis of EEG signals for pain detection applications. For instance, time–frequency domain analysis provides a comprehensive view of how frequency components change over time, which is crucial for capturing transient brain activity [12,37]; however, it can be computationally intensive and may require careful parameter tuning for optimal results [12,40]. On the other hand, frequency features, including various entropy measures and PSD, are effective in quantifying the complexity and energy distribution of EEG signals [10,40], making them useful for distinguishing between different mental states. However, these methods can sometimes overlook temporal dynamics, limiting their effectiveness in rapidly changing brain activities [10].

Wavelet transformation excels at capturing both frequency and temporal information, allowing for the analysis of non-stationary signals; yet, it can be complex to implement and may introduce artifacts if not handled carefully [32]. LEP features, which focus on different peaks in event-related potentials, can highlight specific cognitive processes, but they are sensitive to noise [38]. In contrast, statistical analysis of power bands is straightforward and provides clear insights into the dominant frequencies associated with various cognitive states, but it may simplify the data too much, potentially missing nuanced information, although it is helpful for reducing dimensionality [34]. PCA is valuable for reducing dimensionality and identifying underlying patterns in EEG data, enhancing computational efficiency [37]; however, it may obscure interpretability, as the principal components can be difficult to relate back to original features [37]. Finally, the Choi–Williams distribution is a sophisticated time–frequency representation that captures both time and frequency localization effectively, but its complexity can lead to challenges in interpretation and increased computational load [12,34].

#### 5.5.3. Data Augmentation and Feature Selection

Several studies augmented the feature matrix with additional data. Elsayed et al. [14] introduced an additional layer to the preprocessing phase, which balances the data with new data generated from a noise-affected pain matrix using a VAE. This approach enables nonlinear classification. Leng et al. [40] generated new synthetic feature data mimicking the original EEG data using a Wasserstein GAN with gradient penalty. The synthesized data supplement the insufficient feature data and expand the dataset size. A generator function produces artificial instances from a probability distribution of random noise, and the discriminator strives to differentiate between the generated data and actual data distribution.

**Table 9:** Augmentation and feature selection approaches used in EEG-based pain detection.

Process	Method	Number of Studies	References
Feature Selection	Sequential forward feature selection	2	[10,35]
Data Generation	Autoencoder	1	[14]
	GAN	1	[40]

As shown in the table, some studies applied data augmentation and feature selection methods that can provide significant benefits for BCIs based on EEG signals. However, these methods also have disadvantages, and careful consideration must be given to their implementation and the specific characteristics of the data.

Sequential Forward Feature Selection can effectively identify the most relevant features while reducing dimensionality, which can improve model interpretability and decrease computational load. However, it can be computationally expensive with large datasets [10,35]. On the other hand, data augmentation techniques such as Autoencoders and GANs are valuable for enhancing the robustness of EEG data. Autoencoders can helps to mitigate the issues of limited data availability. This can lead to improved model training and generalization. However, the effectiveness of Autoencoders depends on the quality of the learned representation [14]. Similarly, GANs are powerful generative models capable of producing high-quality synthetic data that can augment training sets, ultimately improving the performance of models. However, training GANs is complex and challenging, often requiring careful tuning and a significant amount of computational resources [40].

#### 5.5.4. Classification

Table 10 summarizes the various ML classifiers employed in previous research. Several studies [12,34,37] detected two pain classes using SVM classifiers, which find the optimal hyperplane separating the two classes. Modares-Haghighi et al. [9] and Zolezzi et al. [36] classified pain levels using multilayered decision trees—SVMs, whereas Afrasiabi et al. [35] employed a binary SVM for binary decision-making at each decision tree node. Leng et al. [40] classified pain levels into five classes using an SVM. Other studies [10,39,40] combined the KNN method for decision-making and classification. Ensemble learning methods based on the SF algorithm or RF algorithm [11,32,40] were also found in the reviewed studies.

Many of the DL-based studies [13,14,44,45,46] utilized neural networks with different architectures and preprocessing approaches (Table 10). Several studies adopted CNNs for pain detection [13,14,45]; others combined CNN and RNN to fuse the spatial and temporal features of pain during EEG signal processing [44]. Fu et al. [46] combined a transformer with a CNN architecture for pain classification. The data are first input to the CNN layer, which extracts the high-level features and reduces the dimensionality of the input. The transformer component of the model (a series of four encoder blocks) then extracts the contextual information from the time-series data.

**Table 10:** Classification approaches used in EEG-based pain detection.

Method	Number of Studies	References
SVM	8	[9,10,12,34,35,36,37,40]
Random forest	3	[11,32,40]
KNN	3	[10,39,40]
CNN	5	[13,14,44,45,46]
RNN	1	[44]
Transformer	1	[46]
LASSO regression	1	[38]

As shown in the table, various classification methods and algorithms used in previous studies exhibit unique characteristics that can influence their performance. The choice of algorithm should take these factors into account to optimize effectiveness in pain detection applications.

For instance, SVM is particularly effective with high-dimensional EEG data, as it can create optimal hyperplanes to separate different mental states and has a strong ability to perform binary classification [9,10]. However, SVM can be computationally intensive and may require fine-tuning of parameters to achieve optimal performance [35]. Moreover, there is a possibility for the overfitting in SVM for unseen data [36]. On the other hand, RF offers the advantage of handling noisy data well and providing insights into feature importance, making it suitable for EEG classification tasks. Its ensemble approach reduces overfitting compared to single decision trees [32], but it may suffer from reduced interpretability and increased computational demands with large datasets. KNN is straightforward to implement and can adapt easily to different EEG patterns without requiring extensive training [10]. However, its performance can degrade with high-dimensional data due to the curse of dimensionality, and it is computationally expensive at prediction time, especially with larger datasets.

CNNs identify spatial patterns in EEG signals, automatically learning relevant features, which enhances classification accuracy and supports nonlinear classification [13,14,45,46]. Nonetheless, CNNs require a substantial amount of labeled training data and can be complex to optimize [13]. Transformers, with their self-attention mechanisms, are gaining traction in processing sequential EEG data, allowing them to capture long-range dependencies effectively [46]. While they provide significant improvements in performance, they demand considerable computational resources and larger datasets for training [46]. Lastly, Lasso regression offers the advantage of feature selection and regularization, which can enhance model interpretability and prevent overfitting [38]. However, it may not capture complex relationships in the data as effectively as more sophisticated models like CNNs or random forests.

## 6. Discussion

This review highlights the evolving landscape of pain detection using EEG through ML and DL methodologies. Both approaches have demonstrated significant potential in accurately classifying pain levels, yet they exhibit distinct strengths and limitations.

The ML studies presented in Table 1 exhibit varying degrees of accuracy, predominantly ranging from 72.7% to 96% across different datasets and methodologies. Notably, methods such as SVM and RF have consistently shown high accuracy rates, with some studies reporting accuracies up to 96% (Zolezzi et al. [36]). These methods are often favored for their interpretability, allowing researchers and clinicians to understand the decision-making process behind classifications. However, their performance is heavily reliant on feature engineering and the quality of the input data. Studies that collected EEG data across diverse stimuli and larger subject pools, such as those by Afrasiabi et al. [35] and Tasci et al. [39], yielded better results, suggesting that data diversity plays a crucial role in enhancing model robustness. In contrast, the DL studies, summarized in Table 2, generally achieved higher accuracy rates, with several studies reporting results above 90%, such as the 97% accuracy achieved by Yu et al. [42] using CNN. This indicates that DL methods, particularly CNNs, are adept at capturing complex patterns in EEG data that traditional ML methods may overlook. For example, the combination of CNN and RNN architectures demonstrated robustness in pain classification, achieving up to 92% accuracy in the study by Wu et al. [44]. The DL models excel in automatically extracting features from raw EEG data, reducing the need for extensive feature selection. This capability allows for the integration of larger datasets and more complex patterns of neural activity, which can be particularly beneficial in clinical settings where data variability is high.

The synthesis of findings from both ML and DL studies reveals a trend toward improved performance with the latter, particularly in studies featuring larger and more diverse datasets. However, the reliance on extensive computational resources and the need for large training datasets can be limiting factors for DL methods, especially in clinical environments where data may be scarce.

Furthermore, both methodologies face challenges related to generalizability. Many studies are conducted on small, homogeneous populations, which may not reflect the broader patient demographic. Future research should aim to validate these models in diverse clinical settings and explore transfer learning approaches to enhance model adaptability. In review, while both ML and DL techniques offer promising avenues for pain detection through EEG, there is a clear need for continued research that not only improves accuracy but also addresses the interpretability and generalizability of these models. By combining the strengths of both approaches and focusing on diverse datasets, future studies can enhance the reliability and applicability of EEG-based pain detection in clinical practice.

### 6.1. Challenges and Limitations

The field of pain detection using EEG data faces several significant challenges that can impact the accuracy and applicability of BCI techniques in pain detection. In our review, we identified some of the challenges and limitations as follows:

Dataset Limitations: Many researchers have resorted to recording their datasets, which are not publicly available for use by other studies. This practice results in variability in data quality, collection methods, and feature representation. Consequently, this fragmented approach limits the comparability of findings across studies and hinders the development of standardized methodologies, making it challenging to test different approaches and draw broader conclusions. Furthermore, numerous studies rely on small sample sizes, often ranging from 10 to 44 subjects. This limited participant pool raises concerns about the generalizability of the findings to broader populations. Additionally, some studies depend on self-reported pain levels collected via questionnaires, which are inherently subjective and may vary significantly between individuals. Feature Engineering and Model Selection: These studies demonstrate inconsistency in the EEG features utilized, with various rhythms and feature extraction methods employed across different research efforts. This variability can lead to challenges in evaluating model performance. Moreover, while some studies compare multiple ML techniques, not all approaches receive equal scrutiny, resulting in gaps in understanding the most effective methods for pain classification.

Accuracy and Reliability: There is a notable variability in reported accuracy rates, ranging from 72.7% to 99%. This inconsistency raises questions about the robustness and reliability of the models developed. Additionally, some studies rely on metrics like the area under the curve (AUC) instead of standardized accuracy measures, which can complicate direct comparisons of performance.

Scalability and Practical Implementation: Translating research findings into real-world clinical applications poses significant challenges. Most studies have not discussed factors such as user acceptance and the complexity of BCI implementation, and the need for larger, publicly available datasets further complicate the path toward effective pain detection solutions.

### 6.2. Future Directions

The findings and insights gained through this pain classification review can guide future investigations and solutions to ongoing challenges in the field. The following key directions are envisaged for future research:

Establishment of Shared Datasets: We identified only two publicly available datasets, namely, selective neural indicators of pain discriminability(https://osf.io/s4ugw accessed on 1 January 2024) and brain mediators of pain (https://osf.io/bsv86 accessed on 1 July 2024). Currently, most researchers independently collect their datasets, which can lead to inconsistencies and hinder reproducibility. The field would substantially benefit from the establishment and maintenance of a shared, publicly available dataset. Such a resource would provide a common foundation for researchers, enabling cross-validation of findings and fostering collaborative research endeavors. By publishing standardized datasets, we can enhance our understanding of pain and accelerate progress in pain assessment, ultimately improving the generalizability and clinical applicability of the findings.

Impact Evaluations of Feature Engineering Strategies: The most informative features in high-dimensional neuroimaging data can be identified using specific feature extraction and selection techniques, but feature extraction is a persistent challenge. Future work should explore the impact of various feature engineering strategies on the performance of pain detection models, possibly by comparing and evaluating the effectiveness of methods that extract features in the time domain, frequency domain, and time–frequency domain. Advanced feature selection and dimensionality reduction techniques must also be explored. A thorough evaluation of feature engineering approaches would enable the development of more robust and accurate pain detection algorithms.

Evaluation of Computational Methods: A number of algorithms and computational methods are used for pain classification. SVMs are effective in high-dimensional spaces and robust to overfitting, making them suitable for complex datasets; however, they can be computationally expensive for large datasets and may struggle with noisy data. KNN is simple to implement and effective for small datasets, but its computational intensity at prediction time and sensitivity to irrelevant features can hinder performance. Random forest enhances generalization by averaging predictions from multiple trees and performs well on large datasets, though it can be less interpretable and resource-intensive. CNN is able to capture spatial hierarchies, but it require large amounts of labeled data and complex designs. Transformers excel in processing sequential data by capturing long-range dependencies through self-attention mechanisms; however, they demand significant computational resources and large datasets, which may limit their applicability for smaller dataset.

Multimodal Data Integration: Future research should attempt to integrate multimodal data; for example, EEG signals could be combined with other physiological signals (e.g., fMRI and heart rate variability) or behavioral data. Such a holistic approach can enhance our understanding of pain mechanisms and comprehensively elucidate a patient’s state, thus improving the accuracy of pain classification.

Real-Time Pain Detection Systems: BCI technology promises the development of real-time pain detection systems that provide immediate feedback to clinicians, allowing timely interventions and personalized treatment strategies. To this end, researchers should focus on optimizing the speed and accuracy of algorithms, ensuring their effective operation in clinical settings.

New technology: Future research should explore the possibility of leveraging multimodal deep learning models such as multimodal large language models and diffusion models. These advanced architectures can enhance the accuracy of pain assessment by analyzing data from various sources, including physiological signals, facial expressions, and patient-reported outcomes. Additionally, the incorporation of wearable devices equipped with advanced sensors can provide continuous monitoring of physiological markers associated with pain, allowing for real-time analysis and timely interventions. Furthermore, exploring the use of diffusion models in generating synthetic data could aid in training robust algorithms, ultimately leading to improved diagnostic tools for pain management.

## 7. Conclusions

The assessment of patients who cannot easily express their pain poses multiple challenges, requiring reliable measurement and determination of pain level and identification of potential treatment processes. BCI applications provide objective insights into the brain activity of patients, such as those who are pregnant, in labor, or paralyzed, potentially improving the pain assessment of such patients.

This paper reviewed the current landscape of pain assessment systems, with a focus on the analysis of EEG signals using traditional algorithms and DL for pain assessment. In addition, we comprehensively analyzed the computational methods and approaches employed in studies related to EEG-based pain detection. This analysis elucidates the diverse techniques utilized in the field, reveals gaps in the existing knowledge, and identifies opportunities for future research.

Our review indicates that DL methods consistently outperform traditional ML techniques in terms of accuracy and predictive power for pain detection using EEG. Specifically, studies show that DL models, particularly CNNs, can leverage large datasets to capture complex patterns in EEG signals that ML methods may overlook.

However, there is significant variability in the methodologies employed across different studies, including the type of EEG equipment used, preprocessing techniques, and classification algorithms. This variability poses challenges for replication and comparison, raising concerns about the generalizability of findings. Many studies are also limited by small sample sizes and a lack of demographic diversity, suggesting the need for larger and more diverse populations in future research.

In conclusion, our review highlights that no single feature extraction or classification technique is universally superior for all applications; the choice of methods is contingent upon the specific system paradigm and task. Therefore, further investigation is necessary to explore the effectiveness of various techniques in different contexts. Collaborative development of datasets can enhance reproducibility and facilitate comprehensive evaluations of algorithm performance, ultimately leading to improved pain assessment systems. 

## Data Availability

No new data were created or analyzed in this study.
